# Is there a role for workplaces in reducing employees' driving to work? Findings from a cross-sectional survey from inner-west Sydney, Australia

**DOI:** 10.1186/1471-2458-10-50

**Published:** 2010-01-31

**Authors:** Li Ming Wen, James Kite, Chris Rissel

**Affiliations:** 1 Health Promotion Service, Sydney South West Area Health Service, Sydney, Australia; 2 School of Public Health, University of Sydney, Sydney, Australia

## Abstract

**Background:**

The role of workplaces in promoting active travel (walking, cycling or using public transport) is relatively unexplored. This study explores the potential for workplaces to reduce employees' driving to work in order to inform the development of workplace interventions for promoting active travel.

**Methods:**

An analysis of a cross-sectional survey was conducted using data from parents/guardians whose children participated in the Central Sydney Walk to School Program in inner-west Sydney, Australia. A total of 888 parents/guardians who were employed and worked outside home were included in this analysis. The role of the workplace in regards to active travel was assessed by asking the respondents' level of agreement to eight statements including workplace encouragement of active travel, flexible working hours, public transport availability, convenient parking, shower and change rooms for employees and whether they lived or worked in a safe place. Self-reported main mode of journey to work and demographic data were collected through a self-administrated survey. Binary logistic regression modelling was used to ascertain independent predictors of driving to work.

**Results:**

Sixty nine per cent of respondents travelled to work by car, and 19% agreed with the statement, "My workplace encourages its employees to go to and from work by public transport, cycling and/or walking (active travel)." The survey respondents with a workplace encouraging active travel to work were significantly less likely to drive to work (49%) than those without this encouragement (73%) with an adjusted odds ratio (AOR) of 0.41 (95% CI 0.23-0.73, P = 0.002). Having convenient public transport close to the workplace or home was also an important factor that could discourage employees from driving to work with AOR 0.17 (95% CI 0.09-0.31, P < 0.0001) and AOR 0.50 (95% CI 0.28-0.90, P = 0.02) respectively. In contrast, convenient parking near the workplace significantly increased the likelihood of respondents driving to work (AOR 4.6, 95% CI 2.8-7.4, P < 0.0001).

**Conclusions:**

There is a significant inverse association between the perception of workplace encouragement for active travel and driving to work. Increases in the number of workplaces that encourage their employees to commute to work via active travel could potentially lead to fewer employees driving to work. In order to make active travel more appealing than driving to work, workplace interventions should consider developing supportive workplace policies and environments.

## Background

Driving to work not only contributes to traffic congestion, air pollution, greenhouse gas emission and lower levels of physical activity [1,2], but also is found to be significantly associated with overweight and obesity in the general population [3,4]. There is a growing body of research indicating that people who are engaged in active travel (walking, cycling or using public transport) are healthier, happier, and have better workplace attendance records [5-7].

Driving to work makes up a significant proportion of all motorised journeys. Over 10 million people are employed in full or part-time work in Australia [8]. Within Sydney, 70% of all journeys to work are by car [9]. Thus, there is a great potential for workplace interventions to promote active travel to work.

However, the role of workplaces in developing policies, facilities and encouragement regarding active travel on employee's modes of journey to work is relatively unexplored. Some research has suggested that there are significant potential benefits that could be gained through workplace policies and interventions which target commuting [7,10-13]. For example, Oja et al reported that over half their respondents were of the opinion that the employer should support employees to commute actively [7]. Despite this, it appears that in both small (under 100 employees) and large companies, employers generally view their role in staff travel as limited to providing parking and that the development of travel plans was not a priority [11].

With increased recognition of the importance of active travel as an effective and sustainable way to protect the environment and promote healthy lifestyles, we implemented the Central Sydney Walk to School Program in 2005-7 [14]. One of the program aims was to promote parents' active travel to work as the study found that children's mode of travel to school is strongly associated with their parents' mode of travel to work [15]. In this current analysis, we explore the potential for workplaces to promote active travel to work.

## Methods

### Design

A cross-sectional self-administered survey was conducted in late 2006 as part of the Central Sydney Walk to School Program. It was approved by the ethics committees of Sydney South West Area Health Service, and the NSW Department of Education and Training. The program was evaluated using a cluster randomised controlled design and the details of the study have been reported elsewhere [14,15].

### Study participants

The Central Sydney Walk to School Program recruited a total of 2232 students and their parents in the study. The students were aged 10-12 (Year 5 and 6) and were from 24 public schools located in the inner west of Sydney. The schools varied in terms of size, socio-economic status, and cultural mix. A letter of invitation with detailed information about the study and questionnaires were distributed to parents via the participating students. The parent/guardian mostly responsible for getting the child to school was asked to complete the survey. A total of 1362 parents completed the survey giving a response rate of 61%. In this current study, we extracted a subset of the data that only included parents/guardians who were employed and did not work from home (n = 888).

### Data collection and measures

Parent mode of transport was classified by responses to the question 'How do you usually get to work in the morning?' to which responses to 'By car?' were 'No' or 'Yes'. Explanatory variables were taken from the following list of questions:

a. My workplace encourages its employees to go to and from work by public transport, cycling and/or walking (active travel)

b. I can work flexible working hours at my workplace

c. There is convenient public transport close to my workplace

d. My workplace has shower and change rooms for its employees

e. There is convenient parking near my workplace

f. The area where I work has a reputation for being a safe place

g. There is convenient public transport close to my home

*h. The area where I live has a reputation for being a safe place*.

Options for response to these questions were on a 5 point scale (strongly agree, agree, neither agree nor disagree, disagree, strongly disagree) and were reduced to a binary form (agree versus don't agree and neither) for the analyses. These questions have been pilot-tested by some parents and guardians and reviewed by research experts in the field, but the reliability and construct validity of these questions have not been tested. Other demographic information including age, gender, and educational level was also collected from the parent. The distance to work was estimated by asking parents, "how far is your work from home?"

### Analysis

All analysis was conducted using SPSS (Version 17) [16]. To assess the association between these explanatory variables and driving to work, cross-tabulations were used with a continuity corrected chi-square and odds ratio to measure the unadjusted strength of association. Cross-tabulations were also used to assess the associations between demographic variables and car use and to reduce demographic variables to binary form. A ROC curve was used to calculate the optimal cut-off of 'distance of work from home' in predicting driving to work. The unadjusted strength of association between binary demographic variables and driving to work was similarly estimated using cross-tabulations using continuity corrected chi-square values and odds ratios.

Binary logistic regression modelling was used to ascertain independent predictors of driving to work. A forwards sequential process was used in which predictor variables were tested in the model in order of their unadjusted association with the outcome variable and only predictors with a P value < 0.1 were retained in the model. Once the significant predictor variables were identified, demographic factors were also tested in the model. Adjusted odds ratios (AOR) with 95% confidence intervals were calculated.

To adjust for clustering by school, the within-school intra-class correlation coefficient (ICC) for the outcome (Travel to work by car) was computed using one way analysis of variance. The F value of 4.105 was then used to calculate the design effect of 3.79 and adjustment factor of 1.95. The average cluster size was 37. Wald values and 95% confidence intervals from the logistic regression model were then further adjusted for the design effect and P values were re-estimated using statistical functions in Excel.

### Results

The characteristics of the study population included in the analyses are shown in Table 1. About 80% of the survey respondents were female and two thirds were aged 40 years and over. Almost half of the respondents (47%) lived more than 10 km from their workplace. Sixty nine per cent of parents/guardians drove to work. Forty five per cent can work flexible hours and 36% reported their workplace had showers and change rooms. Sixty three per cent of the respondents reported that there was convenient public transport close to work and a similar percentage (66%) also reported there was convenient parking near their workplace. Only about one fifth (19%) reported that their workplace encourages active travel. In addition, 23% of the respondents reported having only one child in the household and 44% had more than one car in the household.

**Table 1:** Characteristics of parents who were employed and did not work at home (N = 888 parents)

Characteristic		% of sample
Parent gender	Male	19.4
Parent age	> = 40 yrs	67.3
Employment	Part-time	40.8
	Full-time	59.2
Distance to work	> 5 km	70.2
	> 10 km	47.0
English spoken at home		64.0
No. of children in the household	1	23.0
	> 1	77%
No. of cars in the household	0-1	56%
	> = 2	44%
Tertiary education		69.9
Travel to work by car		69.0
Workplace encourages active travel		18.8
Can work flexible hours		45.1
Convenient public transport close to work		62.6
Workplace has shower and change rooms		35.9
Convenient parking near workplace		65.9
Workplace is in a safe area		53.2
Convenient public transport close to home		68.6
Home is in a safe area		52.2

Table S1 (see additional file 1) shows a number of predictors for having driven to work. It presents the crude odds ratios from bivariate cross-tabulation analysis and adjusted odds ratios from the final logistic regression model as well as adjusted P values after adjusting for the clustering design effect. The respondents who reported that their workplace encourages active travel were significantly less likely to drive to work (49%), compared with those whose workplace did not encourage active travel to work (73%), with an adjusted odds ratio (AOR) of 0.41 (95% CI 0.23-0.73) and an adjusted P = 0.002. Convenient public transport close to work or home is also an important factor that could discourage employees from driving to work with an AOR of 0.17 (95% CI 0.09-0.31), adjusted P < 0.0001 and an AOR of 0.50 (95% CI 0.28-0.90), adjusted P = 0.02 respectively.

In contrast, convenient parking near the workplace was positively associated with driving to work. Compared with those without convenient parking near their workplace, respondents with convenient parking were significantly more likely to drive to work with an AOR of 4.56 (95% CI 2.80-7.43), adjusted P < 0.0001.

Other factors including age, language spoken at home and perception of neighbourhood safety have a weaker but significant association with driving to work. In addition, gender, education level and employment status of parent, as well as number of cars and children in the household were not found to be associated with driving to work in this study.

### Discussion

Our results indicate that there are fewer car journeys to work among those employees who perceive that their workplace encourages active travel. Within our study population, just one fifth of workplaces encourage their employees to travel to and from work by active travel. It is likely that if more workplaces encouraged active travel to work there would be a decrease in driving to work, with consequent benefits of increased physical activity, less congestion and less greenhouse gas production. It should be noted that a strong association was found despite the fact that our participants were parents or guardians of primary school children and therefore likely to have to include dropping off or picking up children from school on their way to and from work.

There have been only a relatively limited number of active transport interventions in Australia, with mixed results. These include the Travelsmart Program in Perth, [17] the Travel Blending Trial in Adelaide [18], the National Walk to Work Day [19] and National Ride to Work Day [20]. All these interventions were predominately focused on individual behaviour changes, including 'walk' or 'ride to work' events, social marketing, and media campaigns. In one international review of 22 active travel workplace interventions, Ogilvie et al found that targeted behaviour change programs can only be effective in changing the transport choices of motivated subgroups [21]. Merom et al argue that it is inactive individuals who are the least likely to adopt or maintain active travel but that it is these individuals who stand to gain the most from switching to active travel [12]. It is clear that targeting individual behaviour will not be sufficient to greatly increase active travel; where possible, interventions should also target organisational changes (i.e. policy and physical environment) in order to bring about a significant shift in transport mode among employees.

Workplaces can encourage active travel by making it easy for employees to walk, cycle or use public transport [22]. Obviously, the capacity for workplaces to support active travel will vary significantly depending on their size and available funding. For instance, the capacity of large corporations or government agencies with thousands of employees is likely to be significantly greater than the local corner shop. Given this fact, the options available for encouraging active travel could vary from increasing awareness of public transport options to allowing employees to purchase long-term public transport tickets through salary deduction, to providing secure, well-lit and sheltered bicycle parking and end-of-trip facilities such as shower, changing and locker facilities. Though not significant in our study, flexible working times are also worthy of consideration as there is some evidence that they encourage active travel amongst employees [23].

Disincentives for car use are also likely to be effective. Some studies have reported that transport behaviour could potentially be influenced by workplace policies that increase the cost of and limit the provision of parking [10,12,13]. Notably, Kingham et al found that providing company cars and fuel for private use greatly increased the likelihood of commuting via car because the true costs of the car are not borne by the user [11]. It follows that reducing the provision of company cars and fuel would encourage more people to take up active travel. Similarly, given our highly significant results in relation to convenient parking near work, it appears that limiting access to parking could be an effective method of discouraging employees from driving to work. However, the ability of workplaces to influence parking supply may be limited, especially in smaller businesses, and may instead be an issue for local government.

Developing a workplace travel plan which details workplace-specific active transport measures and the methods by which they are implemented, is one approach being taken by some organisations [23,24]. For example, Optus, an Australian telecommunications company, developed a detailed transport strategy that includes, among other things, charging a significant amount for onsite parking, providing real-time displays for local public transport options, and funding footpaths in the local area to encourage walking [24]. Monitoring data collected by the company shows maintenance of a relatively high number of their employees who commute via active travel (approximately 45%) compared to all other employees in the local area (approximately 10%) suggesting that their transport strategy is effective in reducing car use and promoting active travel [25].

Our results lend weight to the importance of workplace policy in determining employees' mode of transport to work. However, as our data were derived from self-administered questionnaires, there is no way of knowing what policies are currently in place and their relative effectiveness. For example, some research suggests that infrastructure provision is important in increasing walking and cycling to work [10,13]. However, it has been argued that the infrastructure improvements alone are not sufficient to encourage car commuters to switch to active travel [26]. Equally, we have no data on the respondent's workplace and are thus unable to assess the workplace's capacity to support active travel. Future studies should explore the capacity for different workplaces to promote active travel, examine what policies are in place, and test the effectiveness of these policies in order to better inform subsequent programs and interventions. Further, that having convenient public transport close to home or work had a significant positive association with active travel demonstrates that the workplace is not the only, or even the most important, factor to consider when developing interventions.

### Limitations of this study

There are a number of limitations of the study. The generalisability of the study findings could be limited due to the locality of the study area, inner west Sydney, and the study participants in this analysis, a sub-sample of another study [14]. In particular, the proportion of participants driving to work may have been inflated due to the need to drop off or pick up children from school. Equally, the majority of respondents were female, making it difficult to assess whether this pattern is present across both genders or whether there is a stronger association with one or the other. We also cannot attribute causality, given the cross-sectional nature of our study.

The perception that the workplace encourages active travel is only assessed by self-reports and could vary widely regardless of facilities offered by the workplace. Responses to our statement could therefore differ even if participants' workplaces offered the same facilities and policies. However, the perception of the situation is often the main determinant of behaviour. For example, parents may not let their children walk to school because they perceive it to be dangerous regardless of actual risk.

### Implications for workplace active travel interventions

The potential for workplaces to reduce the number of employees' driving to work described in this study has a number of important implications for developing effective workplace interventions to promote active travel to work. Any interventions need to acknowledge the role of the workplace regarding active travel and creating a supportive environment. Assisting employers to develop active travel policies that encourage employees to travel to work by walking, cycling, or public transport, whilst also making car use less attractive, is a promising strategy, as demonstrated by the Optus example. Whilst we acknowledge that a number of other agencies, most notably governments, have greater potential to facilitate large scale change in travel modes, the role that workplaces can play should not be ignored.

## Conclusion

In this analysis, we found that less than one fifth of workplaces are perceived to encourage their employees to go to and from work by active travel, and that this encouragement is significantly and inversely associated with employees driving to work. These results suggest that the workplace has a significant role to play in promoting active travel to work. In order to be more effective, any active travel interventions should involve the workplace and encourage organisational changes, not just behavioural, that make it easier for employees to commute via active travel.

## Competing interests

The authors declare that they have no competing interests in this study.

## Authors' contributions

LMW conceived the idea of this study and undertook data analysis and interpretation and wrote the original draft. JK assisted in literature review and contributed to writing up this manuscript. CR contributed to writing up this manuscript. All authors have read and approved the final manuscript.

## Pre-publication history

The pre-publication history for this paper can be accessed here:

http://www.biomedcentral.com/1471-2458/10/50/prepub

## Supplementary Material

Additional file 1 **Table S1**. Predictors for driving to work among the 888 parents who were employed and did not work at home using binary logistic regression modelling. Click here for file
