# Supplementary material for: Is there a role for workplaces in reducing employees' driving to work? Findings from a cross-sectional survey from inner-west Sydney, Australia
Source: BMC Public Health. 2010 Jan 31;10:50. doi: 10.1186/1471-2458-10-50 (PMC2825221; doi:10.1186/1471-2458-10-50)
Supplement: Additional file 1 — Table S1. Predictors for driving to work among the 888 parents who were employed and did not work at home using binary logistic regression modelling. [file 1471-2458-10-50-S1.DOC]

**Table 2: Predictors for driving to work among the 888 parents who were employed and did not work at home using binary logistic regression modelling**

| **Predictor** | **Response** | **% who use a car** | **Unadjusted OR (95%CI)*** | **P value** | **AOR**  **(95% CI)**** | **Adjusted P value***** |
| --- | --- | --- | --- | --- | --- | --- |
| **Workplace encourages active travel** | Don’t agree  Agree | 73.3  48.8 | 1  0.35 (0.24, 0.49) | <0.0001 | 0.41 (0.23, 0.73) | 0.002 |
| **Can work flexible hours** | Don’t agree  Agree | 71.6  65.6 | 1  0.76 (0.57, 1.01) | 0.07 | 0.83 (0.51-1.11) | 0.09 |
| **Convenient public transport close to work** | Don’t agree  Agree | 89.3  56.6 | 1  0.16 (0.11, 0.23) | <0.0001 | 0.17 (0.09, 0.31) | <0.0001 |
| **Workplace has shower and change rooms** | Don’t agree  Agree | 69.1  68.5 | 1  0.97 (0.72, 1.31) | 0.91 | - | - |
| **Convenient parking near workplace** | Don’t agree  Agree | 47.7  79.8 | 1  4.35 (3.20, 5.90) | <0.0001 | 4.56 (2.80, 7.43) | <0.0001 |
| **Workplace is in a safe area** | Don’t agree  Agree | 68.2  68.7 | 1  1.03 (0.71, 1.49) | 0.97 | - | - |
| **Convenient public transport close to home** | Don’t agree  Agree | 78.9  64.3 | 1  0.48 (0.34, 0.67) | <0.0001 | 0.50 (0.28, 0.90) | 0.02 |
| **Home is in a safe area** | Don’t agree  Agree | 66.0  71.6 | 1  1.29 (0.97, 1.72) | 0.09 | 1.68 (1.03, 2.75) | 0.04 |
| **English spoken at home** | No  Yes | 63.4  72.2 | 1  1.50 (1.12, 2.00) | 0.01 | 1.69 (1.04, 2.75) | 0.04 |
| **Distance to work > 5 Km** | No  Yes | 67.2  69.8 | 1  1.13 (0.83, 1.54) | 0.48 | - | - |
| **Distance to work >10 Km** | No  Yes | 68.6  69.5 | 1  1.05 (0.79, 1.39) | 0.81 | - | - |
| **Parent age >=40 yrs** | No  Yes | 61.8  72.7 | 1  1.64 (1.22, 2.22) | 0.001 | 1.77 (1.08, 2.90) | 0.02 |
| **Parent gender-male** | No  Yes | 68.9  70.1 | 1  1.08 (0.73-1.28) | 0.75 | - | **-** |

| **Tertiary education** | No  Yes | 70.9  69.3 | 1  0.93 (0.66, 1.31) | 0.74 | - | - |
| --- | --- | --- | --- | --- | --- | --- |
| **Full-time employment** | No  Yes | 70.8  69.7 | 1  0.95 (0.70, 1.28) | 0.79 | - | - |
| **No. of children in the household >1** | No  Yes | 68.2  70.5 | 1  1.12 (0.79, 1.59) | 0.51 | - | - |
| **No. of cars in the household**  **>=2** | No  Yes | 66.3  69.2 | 1  1.16 (0.86, 1.55) | 0.31 | - | - |

* from bivariate cross-tabulation analysis; ** from binary logistic regression with P value and 95% confidence intervals; ***adjusted for design effect
